# 2080: Therapeutic potential of mesenchymal stromal cells for hypoxic ischemic encephalopathy: A systematic review of preclinical studies

**DOI:** 10.1017/cts.2017.198

**Published:** 2018-05-10

**Authors:** Alvaro Moreira, Jamie Archambault, Dawn McDaniel, Peter Hornsby

## Abstract

OBJECTIVES/SPECIFIC AIMS: To assess the efficacy of exogenous administration of MSCs in animal models of HIE. METHODS/STUDY POPULATION: Adhering to the Systematic Review Protocol for Animal Intervention Studies, a systematic search of English articles was performed using MEDLINE, Web of Science, CINAHL, and Google Scholar. Search term items included mesenchymal stem/stromal cell, hypoxic ischemic encephalopathy, asphyxia, cerebral ischemia, and neonatology. We selected randomized and nonrandomized studies that examined in vivo neonatal models of induced HIE. We excluded studies that combined MSCs with an adjunct therapy. Data were collected on study specifics, MSC characteristics, and outcome measurements. The primary outcome was efficacy of MSC treatment, assessed by functional neurologic measures (cognitive, motor, sensory). Risk of bias was assessed using the SYRCLE’s risk of bias tool and we used the ARRIVE guidelines to describe the quality of study reporting. RESULTS/ANTICIPATED RESULTS: A total of 17 preclinical publications focusing on MSC therapy for HIE met our inclusion criteria. Fifteen of the studies (88%) induced HIE in rodents by ligating the common carotid artery followed by a period of hypoxic exposure. Nine (53%) studies derived their MSCs from rodent bone marrow, whereas the other investigators provided xenografts from human bone marrow or umbilical cord-derived MSCs. Range of MSC dose was between 0.25 and 3.5× 106 cells with 71% of the experiments transplanting the MSCs intranasally or intracerebral. Three studies (18%) administered multiple doses. The cylinder rearing test was the most common (73%) sensorimotor functional outcome performed in the first month following the establishment of HIE. All studies demonstrated a reduction in asymmetrical paw preference after receiving MSC therapy. Lesional size was assessed, using neuroimaging or histologic evaluations, and the majority of studies showed a decreased insult following MSC therapy. DISCUSSION/SIGNIFICANCE OF IMPACT: MSC treatment demonstrates improved functional and structural outcomes that are encouraging for future translational studies.

